# Glycerol Monolaurate (GML) and a Nonaqueous Five-Percent GML Gel Kill *Bacillus* and *Clostridium* Spores

**DOI:** 10.1128/mSphereDirect.00597-18

**Published:** 2018-11-21

**Authors:** Patrick M. Schlievert, Samuel H. Kilgore, Gabriela M. Kaus, Theresa D. Ho, Craig D. Ellermeier

**Affiliations:** a Department of Microbiology and Immunology, University of Iowa, Iowa City, Iowa, USA; Antimicrobial Development Specialists, LLC; University of Arkansas for Medical Sciences; University of Idaho; NIAID/NIH

**Keywords:** *Bacillus*, *Clostridium*, endospores, glycerol monolaurate

## Abstract

Glycerol monolaurate is a broadly antimicrobial fatty acid monoester, killing bacteria, fungi, and enveloped viruses. The compound kills stationary-phase cultures of Bacillus anthracis, suggesting that the molecule may kill spores. In this study, we examined the ability of glycerol monolaurate alone or solubilized in a nonaqueous gel to kill vegetative cells and spores of aerobic B. anthracis, B. subtilis, and B. cereus and anaerobic Clostridium perfringens and *Clostridium* (*Clostridioides*) *difficile.* Glycerol monolaurate alone was bactericidal for all five organisms tested. Glycerol monolaurate alone was effective in killing spores. When solubilized in a nonaqueous gel, the glycerol monolaurate gel was bactericidal for all spores tested. The data suggest that glycerol monolaurate nonaqueous gel could be effective in decontaminating environmental and body surfaces, such as skin.

**IMPORTANCE** *Bacillus* and *Clostridium* spores are known to be highly resistant to killing, persisting on environmental and human body surfaces for long periods of time. In favorable environments, these spores may germinate and cause human diseases. It is thus important to identify agents that can be used on both environmental and human skin and mucosal surfaces and that are effective in killing spores. We previously showed that the fatty acid monoester glycerol monolaurate (GML) kills stationary-phase cultures of Bacillus anthracis. Since such cultures are likely to contain spores, it is possible that GML and a human-use-approved GML nonaqueous gel would kill *Bacillus* and *Clostridium* spores. The significance of our studies is that we have identified GML, and, to a greater extent, GML solubilized in a nonaqueous gel, as effective in killing spores from both bacterial genera.

## Abstract (precis)

*Bacillus* and *Clostridium* spores are known to be highly resistant to killing, persisting on environmental and human body surfaces for long periods of time. In favorable environments, these spores may germinate and cause human diseases. It is thus important to identify agents that can be used on both environmental and human skin and mucosal surfaces and that are effective in killing spores. We previously showed that the fatty acid monoester glycerol monolaurate (GML) kills stationary-phase cultures of Bacillus anthracis. Since such cultures are likely to contain spores, it is possible that GML and a human-use-approved GML nonaqueous gel would kill *Bacillus* and *Clostridium* spores. The significance of our studies is that we have identified GML, and, to a greater extent, GML solubilized in a nonaqueous gel, as effective in killing spores from both bacterial genera.

## INTRODUCTION

*Bacillus* and *Clostridium* species produce spores as nutrient levels become limiting; these spores are highly resistant to changes in environment conditions and can withstand both heat and drying. For example, Bacillus subtilis is nonpathogenic, but its spores may contaminate environmental surfaces (1), including workbenches in laboratories and ventilation systems. In contrast, B. anthracis and B. cereus may be environmental contaminants, but these organisms are also important causes of human diseases, such as anthrax (2–4) and necrotizing fasciitis (5, 6), respectively. Similarly, there are large numbers of clostridial species, the majority of which can cause human diseases if introduced to traumatized tissue, for example, Clostridium perfringens (7, 8). Also, treatment of humans with antimicrobials that disrupt the normal microbiota can allow germination and growth of pathogens such as *Clostridium* (*Clostridioides*) *difficile* (9–11). Spores from C. difficile may in turn contaminate the environment and skin and clothing after elimination from the infected host.

Glycerol monolaurate (GML) is a broad-spectrum antimicrobial with large numbers of bacterial targets (12–14). For example, Staphylococcus aureus has 16 two-component systems, all of which appear to be targeted for inactivation by GML (13). GML likely inserts into the plasma membranes of bacteria, with the net effect of preventing structural changes in membrane proteins required for their activity (13). The final effect may be to reduce the potential difference across the plasma membrane, comparably to another broadly antimicrobial molecule, reutericyclin (15). As with reutericyclin, bacteria with an gene conferring immunity to reutericyclin, such as some lactobacilli, are resistant to GML, and, indeed, GML serves as a growth stimulant for such microbes (14).

GML alone is active against most Gram-positive bacteria, such as streptococci and staphylococci, but the molecule is completely inactive against Enterobacteriaceae and Pseudomonas aeruginosa, due to the presence of the intact lipopolysaccharide (13, 16). Gram-negative bacteria with lipo-oligosaccharide, such as *Neisseria,* are susceptible to killing by GML (13). However, all Gram-negative bacteria, as well as most Gram-positive organisms, are highly susceptible to GML when the compound is solubilized in a nonaqueous gel (12, 14, 17, 18). The nonaqueous gel has been used extensively on human, animal, and environmental surfaces (12, 14, 17, 18). A large number of *in vitro* and *in vivo* experiments have shown that 5% GML plus a nonaqueous gel (5% [50,000 µg/ml] GML gel) is both effective at killing bacteria and safe for use on human and animal mucosal and skin surfaces (12, 14, 17, 18).

To date, no studies have assessed the effectiveness of GML alone or of 5% (50,000 µg/ml) GML gel against *Bacillus or Clostridium* spores. The goal of this study was to assess the effectiveness of GML in reducing and eliminating spores. Our studies showed that GML alone was effective in killing vegetative cells of B. subtilis, B. anthracis, C. perfringens, and C. difficile. Additionally, GML alone also killed spores by these same organisms but was not as effective as GML gel. GML gel was effective in killing both vegetative cells and spores. Because of its safety record, 5% (50,000 µg/ml) GML gel may be useful in environmental and human surface contamination with bacterial spores.

## RESULTS

### Effect of GML alone on *Bacillus and Clostridium* vegetative cells and spores.

GML is broadly antimicrobial and prevents exotoxin synthesis, with greatest effect on Gram-positive bacterial species and on Gram-negative species without an intact Enterobacteriaceae lipopolysaccharide, for example, *Neisseria* (13).

**FIG 1:**
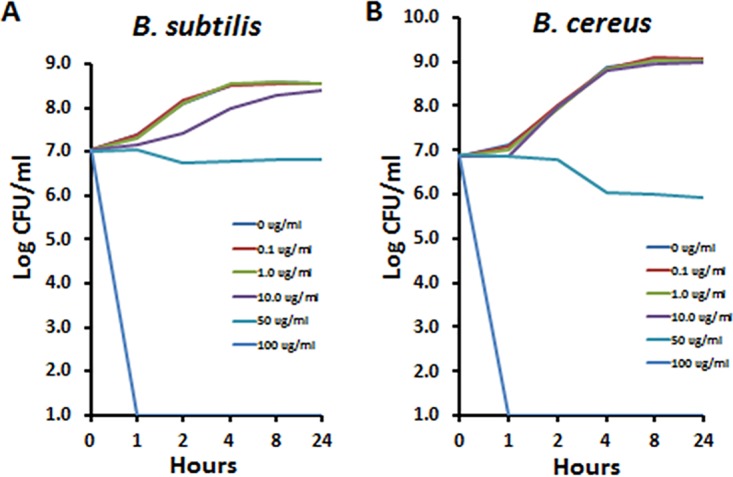
Effect of glycerol monolaurate (GML) on growth of B. subtilis (A) and B. cereus (B). GML concentrations ranged from 0 to 100 µg/ml. The starting inoculum was approximately 10^7^ CFU/ml for each organism. Cultures were aerated by shaking at 200 rpm with samples removed at 0, 1, 2, 4, 8, and 24 h for plate count determination.

We first evaluated the effect of a range of GML concentrations on growth of B. subtilis and B. cereus, noting that stock cultures likely contained both vegetative cells and spores, as we initiated experimentation from 24-h subcultures. Over the 24-h test period, GML alone was bactericidal at the GML concentration of 100 µg/ml for both B. subtilis and B. cereus (Fig. 1). This concentration of GML alone is at the solubility limit of GML in aqueous solutions. GML was bacteriostatic at the 50 µg/ml concentration and did not inhibit growth at lower concentrations. These data indicate that vegetative cells were killed but also that indicate the spores which were likely present in the starting inoculum were either killed or prevented from germinating. The same assay had been previously published for B. anthracis Sterne (19) and so was not repeated.

**FIG 2:**
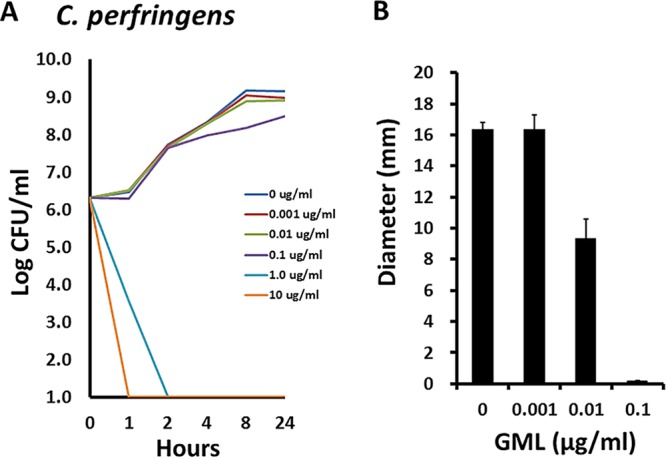
Effect of glycerol monolaurate (GML) on growth of C. perfringens (A) and hemolysin production (B). GML concentrations ranged from 0 to 10 µg/ml. The starting inoculum was approximately 10^7^ CFU/ml. Cultures were incubated to the stationary phase, with samples removed at 0, 1, 2, 4, 8, and 24 h for plate count determination. Lecithinase/hemolysin production was measured only at the 24-h time point as diameter (millimeters) corresponding to rabbit red blood cell lysis. Thin bars in panel B indicate standard deviations.

A similar assay was performed on C. perfringens. For this organism, the minimum bactericidal concentration was 1 µg/ml (Fig. 2A), 2 logs below that for all three *Bacillus* species. At a 10-fold GML concentration below the growth inhibition level (0.1 µg/ml), lecithinase/hemolysin production by C. perfringens was completely inhibited as tested at the 24-h culture time point (Fig. 2B). At concentrations of GML of ≤0.01 µg/ml, inhibition of exotoxin production was partially lost, and at concentrations of GML of <0.01 μg/ml, inhibition of exotoxin production was completely lost.

**FIG 3:**
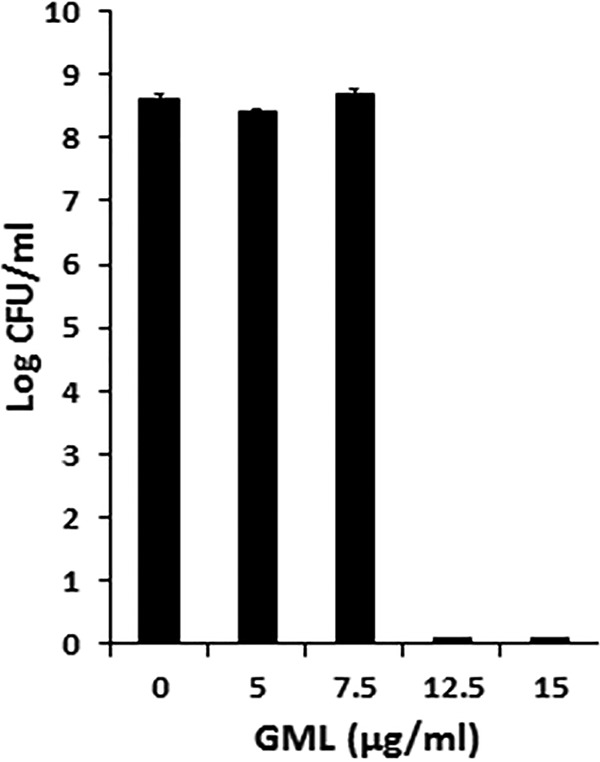
Effect of glycerol monolaurate (GML) on growth of C. difficile R20291. GML concentrations ranged from 0 to 15 µg/ml. The starting inoculum was approximately 10^7^ CFU/ml. Cultures were incubated to the stationary phase, with samples removed at 24 h for plate count determination. Bars indicate standard deviations of 3 plate counts per culture condition.

We did not perform a time course experiment to examine the effect of GML on C. difficile; however, we showed that the minimum bactericidal concentration of GML for this organism was 12.5 µg/ml, well below that for all three *Bacillus* species but closer to that corresponding to the greater sensitivity of C. perfringens (Fig. 3).

**FIG 4:**
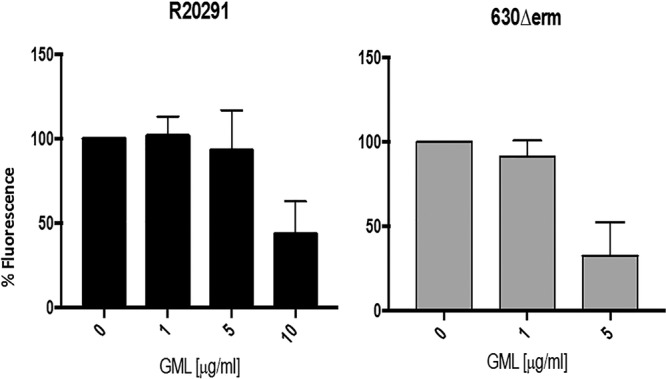
Effect of GML on growth and production of C. difficile toxin A. Data shown are from the highest concentration of GML tested that did not interfere with microbial growth. Effect on P_tcdA_-RFP expression was measured in both strains by analysis of fluorescence intensity compared to the no-GML control, with the no-GML cultures set at 100% fluorescence.

We also tested C. difficile for effects of GML on production of Clostridium difficile toxin A (TcdA) with the use of a fluorescent reporter (red fluorescent protein [RFP]). In this experiment, GML did not interfere with overnight growth at 10 and 5 µg/ml for strains R20291 and 630Δerm, respectively; higher GML concentrations were inhibitory to growth. Production of TcdA was assessed in the two strains at the indicated GML concentrations (Fig. 4). There was an approximately 2-fold reduction in production of TcdA, seen only at the last GML concentration that did not affect C. difficile growth at 10 µg/ml for strain R20291 and 5 µg/ml for strain 630Δerm.

In prior studies, accelerants of activity, for example, a nonaqueous gel as used in the present studies or acidic pH or EDTA, could be added to GML to increase its antimicrobial activity through synergism and/or increasing solubility (13). We have performed many *in vitro* and *in vivo* studies (nonhuman primate and human studies) with 5% (50,000 µg/ml) GML gel (12, 14, 17, 18, 20).

**FIG 5:**
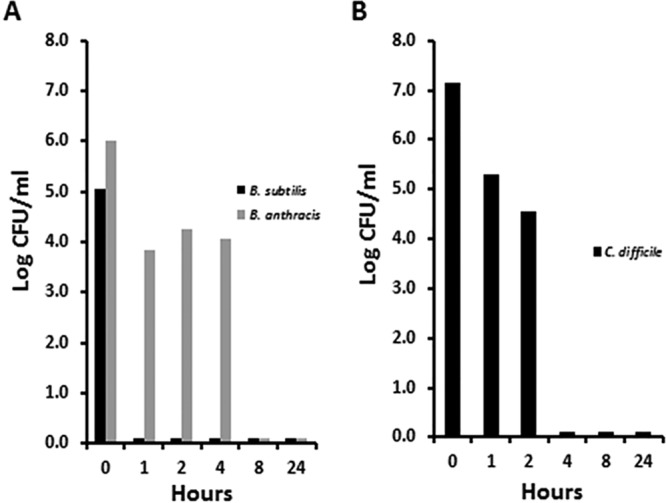
Killing of spores of two *Bacillus* species (A) and C. difficile R20291 (B) by 5% GML gel. The 5% (50,000 µg/ml) GML gel was mixed as 1 volume of bacteria and 9 volumes of GML gel for the indicated time. Plate counts were then performed for determinations of CFU counts per milliliter.

With this as background, we evaluated the ability of the 5% (50,000 µg/ml) GML gel to kill spores of two of the three *Bacillus* species and spores of C. difficile (Fig. 5). The GML gel was bactericidal for three representative spore formers as follows: B. subtilis by 1 h postinoculation, B. anthracis by 8 h postinoculation, and C. difficile by 4 h postinoculation.

**FIG 6:**
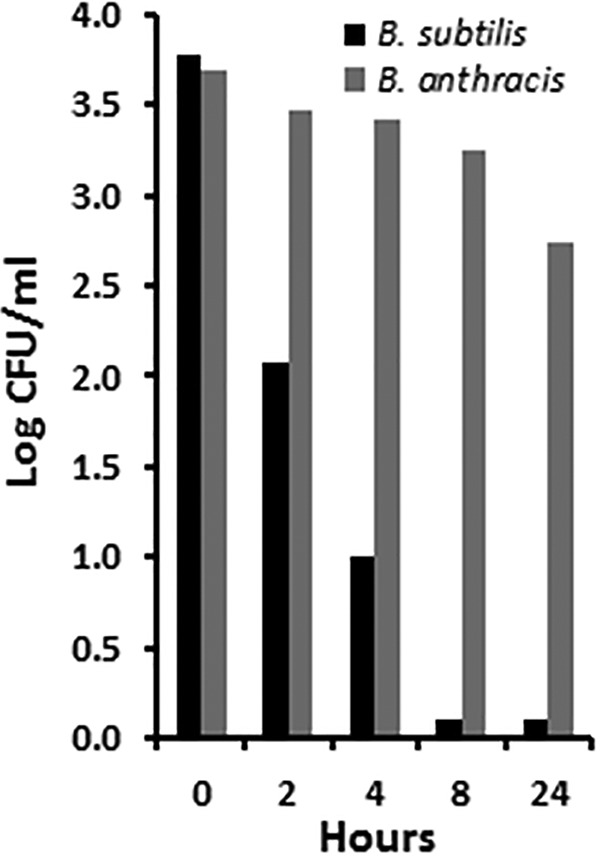
Killing of *Bacillus* spores by 100 µg/ml of glycerol monolaurate (GML) in Todd-Hewitt broth at 37°C through the 24-h test period.

We know the three *Bacillus* strains tested in the studies described above contained spores by the late stationary phase as demonstrated by the results of heat resistance analysis and microscopy with simple spore staining and by the fact that we purchased anthrax spores from Colorado Serum Company. The data corresponding to the figures described above thus imply that GML alone may also be effective in killing spores. This possibility was tested (Fig. 6). GML alone (100 µg/ml) was bactericidal for the B. subtilis spores by 4 h postinoculation. However, the B. anthracis spores were quite resistant to killing, with a log drop of approximately 1 over 24 h.

Our prior studies suggested that one major effect of GML is that of interfering with plasma membrane proteins, such as two-component systems, in vegetative cells, with the final killing effect resulting from dissipation of potential differences across plasma membranes (13). We have also shown that GML prevents biofilm formation and removes established biofilms (13). These are unlikely to be the mechanisms of action in killing spores, as we performed the experiments described above in both nongrowth medium (5% [50,000 µg/ml] GML gel) and growth medium (GML alone added to spores in Todd-Hewitt medium). With this as background, we began studies in attempt to identify why spores are killed by GML.

We first treated B. subtilis spores with GML alone (100 µg/ml) for 2 h and then examined the spores microscopically after staining. We could not see visible signs of spore disintegration compared to non-GML-treated spores as assessed by simple spore staining.

**FIG 7:**
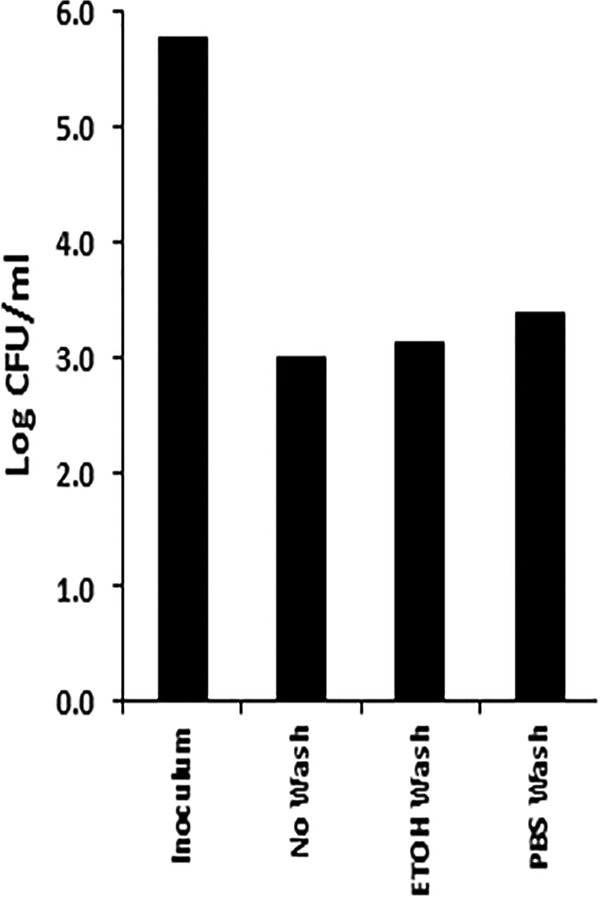
Ethanol (EtOH) washing of B. subtilis spores to remove possibly adherent GML did not increase bacterial viability. The inoculum introduced into 5% (50,000 µg/ml) GML gel was 6.0 × 10^5^ CFU/ml. The ability of spores to germinate and form CFU was determined after GML gel treatment with subsequent EtOH (ETOH Wash) or PBS (PBS Wash) washing or without washing (No Wash).

In a second set of studies, we hypothesized that GML may have been coating the spores, with killing occurring upon subsequent germination. Thus, we treated B. subtilis spores with 5% (50,000 µg/ml) GML gel for 2 h and then washed the spores with absolute ethanol or phosphate-buffered saline (PBS) to solubilize and wash away any surface-attached GML and nonaqueous gel. The starting inoculum of spores was 6.0 × 10^5^/ml. After incubation with GML gel, there was no difference in spore viability (as measured by germination) whether the spores were not rinsed or were rinsed with absolute ethanol or were rinsed with PBS (Fig. 7). Thus, spore killing did not appear to be due to GML gel coating of the spores and killing upon germination.

## DISCUSSION

As noted above, GML alone was bactericidal for vegetative cells of all strains tested. Some of those strains (for example, Bacillus subtilis) contained spores in high concentrations in the stationary phase, suggesting that GML alone and solubilized in a nonaqueous, glycol-based gel might kill resistant spores. Our studies showed that GML alone was bactericidal for vegetative cells of all five organisms, including 3 aerobes and 2 anaerobes. However, 5% (50,000 µg/ml) GML solubilized in the nonaqueous gel (5% [50,000 µg/ml] GML gel) was more effective in killing spores of these same organisms than GML alone. These studies are important since both GML and the nonaqueous gel have been shown to be safe for chronic use in nonhuman primates (12, 17, 18) and human mucosal surfaces (14, 20). This suggests in particular that the 5% (50,000 µg/ml) GML gel could be used to decontaminate environmental surfaces and human skin and mucosal surfaces of C. difficile to help reduce spread of the pathogen in hospitals.

B. anthracis has been used as an agent of bioterrorism, notably as spread through the mail. Our studies suggest that 5% (50,000 µg/ml) GML gel could be used to decontaminate environmental surfaces contaminated with anthrax spores. Our studies showed that these spores were killed only slowly by GML alone at concentrations at the solubility limit in aqueous solutions, unlike spores from B. subtilis. However, the same organisms were completely killed by use of 5% (50,000 µg/ml) GML gel. It is clear from our prior studies that the nonaqueous, glycol-based gel alone, used to solubilize 5% (50,000 µg/ml) GML, has antimicrobial properties (13). Additionally, our prior studies with use of GML alone at concentrations that exceed the solubility limit at 37°C (100 µg/ml) showed that GML has antimicrobial activity for bacteria not killed at the 100 µg/ml solubility limit (16). The latter study suggested that soluble GML may become embedded in the bacterial membrane, thereby removing GML from solution and causing the previously insoluble GML to become soluble, with added membrane insertion, until antimicrobial activity is achieved. In our studies, it is therefore likely that it was the combination of the nonaqueous gel and the greatly increased amount of soluble GML in the gel that allowed greater killing by 5% (50,000 µg/ml) GML gel than by GML alone. Also, other researchers have shown that GML can function in concert with other antimicrobials to increase efficacy (21). It is possible that addition of other agents to GML alone or increased GML amounts in the GML gel would further increase the rate of killing of B. anthracis spores. At the same time, it is worth considering that environmental surfaces are unlikely to have such high concentrations of spores as were present per milliliter in our studies, increasing the likelihood that GML gel alone could be used.

It is unclear why the spores of all organisms were killed by 5% (50,000 µg/ml) GML gel, with Bacillus subtilis alone being highly susceptible to killing by GML without added nonaqueous gel. We performed preliminary studies to test for visible alterations in B. subtilis spores to determine if GML was killing B. subtilis upon germination. The results of both of these studies were negative. Although GML is often thought of as a surfactant, prior studies showed that GML stabilizes rather than destabilizes mammalian cells and bacteria (22, 23). Those prior studies also showed resistance of red blood cells to lysis in the presence of hypotonic solutions containing GML (22) and interference with lipid raft mobility in immune cells (24, 25). It is also possible that GML in some way further stabilizes the spore coats to prevent regeneration.

Previously, it was shown that GML prevents exotoxin production by Gram-positive bacteria at concentrations that do not kill toxin-producing bacteria (16, 19, 23). This was also observed in our studies, in which GML alone at concentrations below those that inhibit growth prevented hemolysin/lecithinase production by C. perfringens. Previously, it was shown that this effect of prevention of exotoxin production resulted in part from GML plasma membrane effects on two-component systems in interference with transcription (23). We do not know the precise effect of GML with respect to prevention of exotoxin production by C. perfringens, but the explanation is most likely to involve effects that increase the rigidity of the plasma membranes and interfere with signal transduction as shown previously. Unlike the results seen with other Gram-positive microbes, we saw only minimal (approximately 2-fold) effects of GML on exotoxin production by C. difficile that were independent of effects on microbial growth.

In sum, our studies have shown that the use of GML alone was effective in killing Gram-positive spore-forming bacteria, with subinhibitory concentrations preventing exotoxin production. A gel composed of 5% (50,000 µg/ml) GML solubilized in glycols and approved for use in humans was effective in killing spores produced by the same microbes.

## MATERIALS AND METHODS

### Bacteria.

B. subtilis, B. cereus, and C. perfringens, kindly provided originally by Dennis W. Watson, University of Minnesota, Minneapolis, MN (now deceased), were cultured from lyophilized Schlievert laboratory stocks. B. anthracis Sterne was obtained from the Colorado Serum Company, Denver, CO. C. difficile R20291 is of ribotype 027 and is maintained in the Ellermeier laboratory (26). The *Bacillus* and C. perfringens strains were cultured in Todd-Hewitt broth or on Todd-Hewitt agar plates. C. difficile was cultured in or on TY broth/agar and plated on CCFA (Anaerobe Systems, CA) plates and incubated at 37°C anaerobically for 24 h before enumeration. *Bacillus* strains were cultured, and plates were incubated aerobically in a standard incubator at 37°C. Clostridium difficile was cultured, and plates were incubated to the stationary phase in a Coy anaerobic chamber at 37°C. C. perfringens was cultured to the stationary phase in GasPak jars (Carolina Biological Supply Company, Burlington, NC) at 37°C, and plates were incubated in the same jars.

For the purposes of this study and as often defined in diagnostic microbiology, we define bactericidal results as represented by a ≥3 log_10_ reduction in CFU compared to non-GML control results and bacteriostatic results as represented by CFU counts within 1 log_10_ in similar comparisons. Our experience with GML and 5% (50,000 µg/ml) GML gel is that the minimum bactericidal concentration and MIC are typically close to each other (13).

### Production of spores.

B. anthracis spores were used as purchased from the Colorado Serum Company. Spores from B. subtilis, B. cereus, and C. perfringens were prepared as follows. The organisms were cultured overnight in 25 ml Todd-Hewitt broth at 37°C. Cells were then collected by centrifugation (4,000 × *g*, 15 min), suspended in 5 ml phosphate-buffered saline (PBS; 0.001 M sodium phosphate [pH 7.2], 0.15 M NaCl), and heated to 80°C for 10 min. The following method (27) was used to produce C. difficile spores. Strain R20291 was grown overnight in tryptone-yeast extract broth at 37°C in a Coy anaerobic chamber with 10% hydrogen, 5% carbon dioxide, and 85% nitrogen. Approximately 0.2 ml of overnight culture was spread on each of 2 SMC plates [9% Bacto peptone, 0.5% proteose peptone, 0.1% (NH_4_)_2_SO_4_, 0.15% Tris base, 1.5% agar] using sterile beads and incubated at 37°C anaerobically for 4 days. Plates were then removed from the anaerobic chamber, and each plate was flooded with 5 ml of sterile PBS. Using a disposable inoculating loop, the bacterial lawns were resuspended. The resuspensions from each plate were pooled and washed in sterile PBS. After centrifugation (3,000 × *g*, 15 min), the pellet was resuspended in 10 ml of 95% ethanol and incubated for 1 h at room temperature. After incubation, spore preparations were washed 3 times in PBS. After the final wash, spore preparations were resuspended in 1 ml of PBS; these preparations were incubated at 70°C for 20 min to kill vegetative cells. Spores were then diluted in PBS and transferred to the anaerobic chamber. Dilutions were plated on CCFA (Anaerobe Systems, CA) plates and incubated at 37°C anaerobically for 24 h before enumeration.

### Glycerol monolaurate (GML) and GML gel.

Food-grade GML was purchased from Colonial Company, Inc., South Pittsburg, TN. A stock solution of the compound was dissolved in absolute ethanol at 100 mg/ml. GML gel was prepared by dissolving 5% (50,000 µg/ml) GML in a nonaqueous gel that consisted of pharmacy-grade reagents in the following: propylene glycol (73.55% [wt/wt]), polyethylene glycol 400 NF (25% [wt/wt]), hydroxypropyl cellulose (1.25% [wt/wt]) (pH 4.5).

### Experimentation with GML alone.

*Bacillus* species were cultured in 25-ml flasks for designated time periods in the presence of various concentrations of GML from a 100,000 µg/ml stock solution. Culture conditions were aerobic, with 200 rpm shaking at 37°C. Samples were removed at indicated times for serial 10-fold dilution plate counts and plated onto Todd-Hewitt agar plates (Difco, Detroit, MI). C. perfringens was cultured to the stationary phase in GasPak jars in 25 ml of Todd-Hewitt broth at 37°C for indicated times. Samples were removed for serial 10-fold dilution plate counts, and broths and plates were immediately returned to the GasPak jars. Samples of Todd-Hewitt broth cultures were also centrifuged (1,000 × *g*, 15 min) and sterilized by filtration (Millex-GS; Merck Millipore Ltd., Tullagreen, Carrigtwohill, County Cork, Ireland) (0.22 μm pore size). Filtrates were used for determination of rabbit red blood cell lysis on 0.85% agarose slides (28). C. difficile was cultured in the presence of indicated concentrations of GML for 24 h in an anaerobic chamber. At that time, samples were removed for plate counts in the same anaerobic chamber. For measurement of the effect of GML on exotoxin production by C. difficile, the following procedures were used. Overnight cultures were subcultured 1:100 in TY medium supplemented with thiamphenicol at 10 µg/ml and with various concentrations of GML. Cultures were grown overnight and fixed as previously described (29). Fluorescence was measured at the Flow Cytometry Facility at the University of Iowa on an LSR II instrument (Becton, Dickinson). Strains and plasmid used for these studies, as described by Ransom et al. (30), included the following: RAN925, which is constructed as R20291/pRAN737(P*_tcdA_*::*rfpcat*), and GMK134, which is constructed as CD630Δerm/pRAN737(P*_tcdA_*::*rfp cat*). Plasmid pRAN737 was a pDSW1728 derivative with P*_tcdA_*::*rfp*.

### Experimentation with GML gel.

Spores (1/10 final volume of spores) were added to 5% (50,000 µg/ml) GML gel at 37°C for indicated periods of time. Samples were removed, and serial dilution plate counts (colony formation) were used for determination of spore germination into vegetative cells. Spore levels in initial cultures were determined by plate counts prior to addition to the GML gel. Experiments with *Bacillus* species were performed aerobically, whereas those with C. difficile were performed in an anaerobic chamber. The GML gel was prereduced 24 h prior to experimentation.

We considered that GML might adhere to spores and in that way kill spores upon germination. To test this, B. subtilis spores, which were the most easily killed by GML gel, were treated with GML gel for 2 h at 37°C. The suspension was then centrifuged (4,000 × *g*, 15 min), and the pellets were resuspended in 1 ml of absolute ethanol to solubilize any GML adherent to spores or in 1 ml of PBS as a negative control. The preparations were again centrifuged, and the pellets were dried and then suspended in Todd-Hewitt broth for plate count determination.

### Statistics.

For some experimental data, standard deviations of the means are presented. Where standard deviations are not presented, experiments were repeated with similar results.
